# Journalistic media and adolescents in the COVID-19 pandemic: a documental analysis

**DOI:** 10.1590/1984-0462/2024/42/2023041

**Published:** 2023-12-22

**Authors:** Marcela Claudia de Paula Oliveira, Emilia Chagas Costa, Lygia Maria Pereira da Silva, Livia Novaes Vieira Barbosa, Mauro Virgilio Gomes de Barros, José Henrique Cavalcanti Mota, Marco Aurelio de Valois Correia

**Affiliations:** a Universidade de Pernambuco, Recife, PE, Brazil.; b Universidade Federal de Pernambuco, Recife, PE, Brazil.; c Universidade Federal Rural de Pernambuco, Recife, PE, Brazil.

**Keywords:** Adolescent, Covid-19, Mental health, Exercise, Quality of life, Adolescente, COVID-19, Saúde mental, Exercício físico, Qualidade de vida

## Abstract

**Objective:**

To analyze how the journalistic media has described the issues of quality of life (QoL), physical activity (PA) and mental health (MH) of adolescents during the COVID-19 pandemic.

**Methods:**

This is a descriptive and qualitative study that used content analysis. Sixty-two journalistic publications were analyzed from a total of 8211 published by the most read newspapers in each Brazilian region between December 2019 and August 2021.

**Results:**

The results were grouped and evaluated in three categories: QoL (n=11), PA (n =9) and MH (n=42). In the analyzed period, the adolescents had more time of exposure to screens, contributing to an inadequate diet, a decrease in PA and impairments in QoL. According to the media publications, the pandemic has also contributed to an increase in anxiety, depression, loneliness and fear resulting from the mental and emotional disorganization caused by the abrupt change in routine. Social vulnerability was presented as an aggravating factor in this context. The journalistic media did not pay the necessary attention to adolescents regarding the negative consequences of the pandemic on QoL, PA and MH.

**Conclusions:**

The analyzed reports showed that the pandemic caused a decrease in social interaction, feelings of uncertainty, fear and the appearance/exacerbation of symptoms of anxiety, stress and depression. Social vulnerability was presented as another obstacle to be faced in this problem.

## RESUMO

**Objetivo:**

Analisar como a mídia jornalística tem descrito as questões de qualidade de vida (QV), atividade física (AF) e saúde mental (SM) de adolescentes durante a pandemia da COVID-19.

**Métodos:**

Trata-se de um estudo descritivo e qualitativo que utilizou a análise de conteúdo. Foram analisadas 62 publicações jornalísticas do total de 8.211 veiculadas pelos jornais mais lidos de cada região brasileira entre dezembro de 2019 e agosto de 2021.

**Resultados:**

Os resultados foram agrupados e avaliados em três categorias: QV (n=11), AF (n=9) e SM (n=42). No período analisado as publicações jornalísticas sugerem que os adolescentes apresentaram maior tempo de exposição a telas, contribuindo para uma alimentação inadequada, diminuição da AF e prejuízos na QV. A pandemia também contribuiu com aumento da ansiedade, depressão, solidão e medo resultantes da desorganização mental e emocional causada pela mudança abrupta de rotina. A vulnerabilidade social apresentou-se como um fator agravante nesse contexto. As mídias jornalísticas não deram a atenção necessária aos adolescentes no que se refere às consequências negativas da pandemia na QV, AF e SM.

**Conclusões:**

As reportagens analisadas mostraram que a pandemia causou diminuição na interação social, sentimentos de incerteza, medo e o aparecimento/exacerbação de sintomas de ansiedade, estresse e depressão. A vulnerabilidade social apresentou-se como mais um obstáculo a ser enfrentado nesta problemática.

## INTRODUCTION

Maintaining a healthy lifestyle associated with physical activity (PA) can be considered an important strategy for improving health indicators and quality of life (QoL) for all ages.^1^ With regard to adolescents, a period marked by intense biological, psychological and social transformations, the maintenance of healthy habits can reduce the risks of possible health problems and contribute to the reduction of morbidities.^1–4^

The COVID-19 pandemic showed that adolescents did not present themselves as a more vulnerable group for infections by the SARS-CoV-2 virus; despite this, several factors linked to the pandemic have a direct negative influence on this population.^4^ Mental health (MH) stands out in this scenario and needs to be monitored in these young people with greater attention, given the concerns related to the transmissibility of the virus, uncertainties about the severity of the disease, the unpredictability regarding the duration of the pandemic and its consequences.^5^

Social isolation was one of the most used strategic measures to control the spread of the virus,^4^ but it had no intentional negative consequences for health, such as increased physical inactivity, anxiety and depression. In addition, it can worsen the mental state of adolescents with pre-existing psychiatric problems. Disorders resulting from MH and QoL in adolescents, if not identified and treated, can accompany the individual for a lifetime.^6,7^

The journalistic coverage of COVID-19 led to a daily approach to the topic in the media, contributing to the dissemination of scientifically based information.^1^ Faced with the phenomenon, the press sought to understand the facts and make them public. However,^1^ no studies were found that evaluated adolescence in this context.

Communication media (television, radio, newspapers) are important means of spreading information and forming public opinion for society.^1^ The media has the power to influence matters of interest, based on the information transmitted more regularly. This article aimed to analyze how the journalistic media has described the issues of QoL, PA and MH of adolescents facing the COVID-19 pandemic in Brazilian newspapers.

## METHOD

This is a qualitative descriptive study using content analysis (CA).^8^ This research uses information in the public domain and is supported by resolution n. 510 of April 7, 2016 (Research Ethics Committee of the National Commission on Research Ethics — CEP/CONEP).

Bardin’s CA^8,9^ is structured in three phases: pre-analysis, material exploration, treatment of results and interpretation. To achieve national coverage, journalistic publications were selected from *online* databases of the three most read newspapers in each Brazilian region, according to the LatAm Journalism Review^10^ (Table 1). The search strategy used the descriptors “adolescent” and “COVID-19”. The inclusion criteria were articles published from December 2019 to August 2021 on the consequences of the COVID-19 pandemic on MH or QoL or PA in adolescents classified according to the World Health Organization (ten to 19 years old).^2^ The excerpts selected to compose the analysis and discussion of the data were products obtained from the opinion of journalists and/or interviewees, as well as clippings from scientific texts and expert opinions.

**Table 1:** Mapping of the three most read newspapers in the Brazilian Regions.^10^

North Region	Northeast Region	Midwest Region	South Region	Southeast Region
A Crítica (Manaus)	Jornal do Comércio (Pernambuco)	Correio Brasiliense (Distrito Federal)	Correio do Povo (Rio Grande do Sul)	O Globo (Rio de Janeiro)
O Liberal (Pará)	Diário do Nordeste (Fortaleza)	Correio do Estado (Mato Grosso do Sul)	Diário Gaúcho (Rio Grande do Sul)	Folha de São Paulo (São Paulo)
A Gazeta (Acre)	Correio (Salvador)	Diário da Manhã (Goiânia)	Zero Hora (Rio Grande do Sul)	Estado de São Paulo (São Paulo)

The search and analysis of articles were conducted independently by two evaluators according to previously established strategies, with differences resolved by a third and fourth author and exported to Microsoft Excel 2010. Duplicate publications were identified and removed from the analysis.

## RESULTS

**Figure 1:**
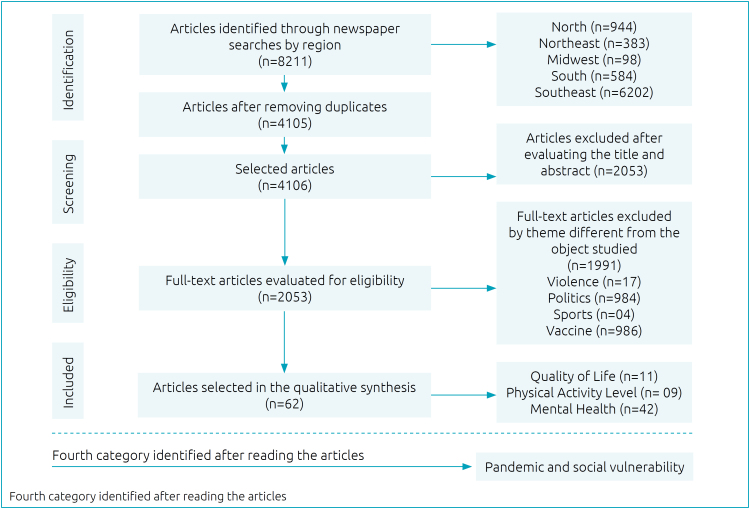
Flowchart of the selection of journalistic publications.

From the cataloging of news (Figure 1), 62 journalistic publications were analyzed out of a total of 8211, which were divided into three categories of analysis. The first deals with reports related to QoL (n=11), where longer exposure time in front of screens and inadequate nutrition was identified. The second deals with PA (n=09) during the pandemic and it was observed that a sedentary lifestyle predominated during the pandemic period. In the third, the MH (n=42) of adolescents selected from social isolation was addressed, also addressing the psychological impacts affected by the adolescent public during the pandemic (Table 2, 3 and 4).

**Table 2:** Characterization of journalistic articles about quality of life in adolescents.

	Report title	Publication newspaper	Subject	Publication date
1	In the pandemic, the study time of adolescents from the upper classes is 64% longer than that of the poor	O Globo	QoL	10/20/2020
2	Education is an essential activity	A Folha	QoL	01/26/2021
3	Virtual environment guarantees the realization of Young Entrepreneurial Apprentice classes	Correio	QoL	02/15/2021
4	How the mess in the school year impacts the entry of young people into universities and the job market	Correio	QoL	05/09/2021
5	COVID: number of children and adolescents infected in 2021 is greater than in 2020 as a whole	Correio	QoL	06/04/2021
6	Preventive measures are little adopted by children, says study	A Gazeta	QoL	07/16/2021
7	“Sensation of freedom” and “back to school”: what vaccinated adolescents with comorbidities say in Porto Alegre	Zero Hora	QoL	07/22/2021
8	Secretary of Health rebuts critics of the return to face-to-face classes in Espírito Santo	Correio	QoL	01/08/2021
9	Squares and parks register a large movement of visitors on the last weekend of the holidays	A Crítica	QoL	01/08/2021
10	Face-to-face semester is monitored to prevent COVID-19	A Crítica	QoL	02/08/2021
11	Seven out of ten ophthalmologists report high myopia in children and adolescents	A Crítica	QoL	02/08/2021

QoL: Quality of Life.

**Table 3:** Characterization of journalistic articles about physical activity level in adolescents.

	Report title	Publication newspaper	Subject	Publication date
1	In the pandemic, 1/3 of families with children and young people ate more industrialized foods	Zero Hora	PAL	08/25/2020
2	Study on obesity makes warning regarding children and adolescents in social distancing	Zero Hora	PAL	08/27/2020
3	Physical education in times of a pandemic, how and why do it?	Estado	PAL	11/17/2020
4	COVID-19: teenagers report sadness, sedentary lifestyle and difficulties sleeping and taking classes during the pandemic	O Globo	PAL	12/01/2020
5	World Health Day: Obesity deserves systemic treatment	Estado	PAL	04/07/2021
6	The pandemic calls attention to obesity treatments	Estado	PAL	04/07/2021
7	Feeding children worries families on vacation and during the pandemic	Estado	PAL	07/23/2021
8	From 5% to 8% of children and adolescents in Pará are obese	A Crítica	PAL	01/08/2021
9	Pandemic raises cases of anxiety and high cholesterol in children and adolescents	O Globo	PAL	04/08/2021

PAL: Physical Activity Level.

**Table 4:** Characterization of journalistic articles about mental health in adolescents.

	Report title	Publication newspaper	Subject	Publication date
1	Nothing will be like before: 10 changes that the pandemic has already brought about in your life	Correio	MH	04/26/2020
2	With schools closed during isolation, psychologists and pedagogues treat children and adolescents over the internet	Estado	MH	05/29/2020
3	UNESCO: the pandemic exacerbates the exclusion of children and young people from education in the world	Correio Brasiliense	MH	06/23/2020
4	Depression increases among children and adolescents during the pandemic	O Globo	MH	06/30/2020
5	Pandemic and social distancing challenge mental health	Correio do Povo	MH	07/20/2020
6	Toxic stress affects children and adolescents in the pandemic	Estado	MH	07/20/2020
7	In the pandemic, 1/3 of families with children and young people in Brazil ate more industrialized foods	Estado	MH	08/25/2020
8	Adolescents and young people drop out of school in the pandemic	Estado	MH	08/28/2020
9	Teen mental health worries doctors and families with the pandemic	Estado	MH	09/14/2020
10	With eating disorders, appearance can change	Estado	MH	09/21/2020
11	Nine out of ten girls suffer from anxiety due to the coronavirus pandemic, research says	Estado	MH	09/25/2020
12	We need to talk about unwanted teen pregnancy	Estado	MH	09/30/2020
13	New wave of the pandemic is to villainize young people	Folha	MH	11/26/2020
14	COVID-19: teenagers report sadness, sedentary lifestyle and difficulties sleeping and taking classes during pandemic	O Globo	MH	12/01/2020
15	Vulnerable children and adolescents in the pandemic	O Globo	MH	12/11/2020
16	Games: the ‘escape valve’ for children and teenagers	Zero Hora	MH	01/31/2021
17	The Anxiety Pandemic	Estado	MH	02/27/2021
18	The pandemic has delayed child development and threatens a generation, says Unicef	Zero Hora	MH	03/11/2021
19	The pandemic increases cases of anxiety and high cholesterol in children	A Gazeta	MH	04/04/2021
20	COVID-19 and physical isolation create warning about childhood depression	Correio Brasiliense	MH	04/11/2021
21	The impact of the pandemic on young people and adolescents	Estado	MH	04/22/2021
22	Young people face ‘cage syndrome’	Zero Hora	MH	04/25/2021
23	The pandemic impacts mental health of mothers, who show signs of stress and depression	Estado	MH	05/03/2021
24	More than 77 thousand students stopped studying in Espírito Santo due to the pandemic	A Gazeta	MH	05/05/2021
25	Pandemic stress leads to bruxism and even tooth fracture	A Gazeta	MH	05/20/2021
26	School dropout is higher in states without face-to-face classes in the pandemic	A Gazeta	MH	05/31/2021
27	Teenagers are in crisis. And your parents too	Estado	MH	05/30/2021
28	Four out of ten students thought about quitting their studies due to the pandemic	Diário da Manhã	MH	06/14/2021
29	Four out of ten students thought about quitting their studies due to the pandemic, says Conjuve	Correio	MH	06/14/2021
30	Five facts about the impact of the pandemic on childhood in Brazil	Estado	MH	06/17/2021
31	Pandemic vacation with preteens	Estado	MH	07/13/2021
32	It’s not a pandemic effect: mental health was already a public health problem and it’s to all of us	Folha	MH	07/14/2021
33	A year and a half of a pandemic on the couch	Estado	MH	07/14/2021
34	Teenage suicide in Uruguay increased by 45% in 2020	Diário Gaúcho	MH	07/16/2021
35	Mental health challenges in Brazil	Estado	MH	07/20/2021
36	Mental health with the return to face-to-face classes	Estado	MH	07/22/2021
37	Study shared by Damares does not attribute child suicides to isolation in the pandemic	Jornal do Comércio	MH	07/22/2021
38	Without schools, children increased their time on television and video games during the pandemic, says Datafolha	O Globo	MH	07/30/2021
39	Back to face-to-face classes a year and a half later: students and families are torn between joy and anxiety	O Globo	MH	01/08/2021
40	With the pandemic, 44% of children and adolescents felt sadder	Diário da Manhã	MH	08/08/2021
41	The pandemic aggravates the situation of aggression against minors	Diário da Manhã	MH	08/15/2021
42	COVID sequelae in children: hospitals treat heart problems, anxiety and fatigue	Estado	MH	08/24/2021

SM: Mental Health.

The need to include a fourth category entitled pandemic and social vulnerability was identified. It was chosen considering that adolescents are represented in different ways in society. In this category, reports focused on the experience of the pandemic were identified, showing that the rich and poor do not experience the same effects of the pandemic.

## DISCUSSION

### Quality of life of adolescents during the pandemic

In this category, 11 journalistic stories were identified (Table 2) about the different changes in the lifestyles of Brazilian adolescents during social distancing resulting from the COVID-19 pandemic. The changes observed were an increase in the consumption of fast and unhealthy foods, a reduction in outdoor activities, reduced peer interaction and sedentary behavior.

QoL can be influenced by several factors and is related to aspects of health, self-esteem, relationships with family, friends, school and work. Adolescents who spent a large part of the day at school were restricted to the home environment full-time. In addition, social distancing may have had repercussions on family relationships, socialization and development.^9^

*The research shows that families with young people aged 0 to 17 are more vulnerable during the pandemic in all scenarios analyzed and states that children and adolescents are “hidden victims of the pandemic”*. (Diário Gaúcho, Rio Grande do Sul, Brazil, 08/25/2020 — Study on obesity warns about children and adolescents in social distancing).

It is believed that social distancing was one of the most efficient measures to prevent the spread of the disease and reduce cases of COVID-19.^11^ However, it had direct repercussions on the adolescents’ QoL. In this context, these adolescents’ QoL may be compromised as they showed changes in eating, activity and sleep behavior in just three weeks of confinement.^9,11^

With regard to education in times of a pandemic, few reports observed that adolescents considered school activities in remote teaching easy and found the classes very interesting,^9^ as shown in an excerpt from the newspaper *Correio* (2021): “I was amazed by the boys, they participated a lot in the discipline, brought experiences that they had already had with reading a book and movie”, and 73.3% of adolescents^9^ showed concern and fear of getting low grades or failing the school year. This shows that technology, previously viewed as something that took the teenager out of social life, became increasingly used during isolation.

In the pandemic scenario, internet consumption has increased the digital sociability of this population, considering *online classes* and the maintenance of relationships through social networks, games and applications.^9^ Cell phones cannot be considered only as a source of entertainment, but as a tool that can help the educational process and virtual interaction with peers.^12^ Another issue to consider is that the predominance of screen time on electronic devices can cause dry eye syndrome or computer visual syndrome.^13^

*“In addition to classes on the computer, he plays with the tablet, with the cell phone. What we do is try to limit the use, but during the pandemic this is even more complicated”*. (O Estado de São Paulo, São Paulo, Brazil, 08/02/2021 — Seven out of ten ophthalmologists report high myopia in children and adolescents).

*Children following exactly the same patterns that I observed in the pre-pandemic period*. (Zero Hora, Rio Grande do Sul, Brazil, 01/31/2021 — Games: the ‘escape valve’ for children and adolescents)

The average time of screen exposure is longer than the recommended^4,12^ and should not exceed two hours a day,^14^ with educational content appropriate for the age group. This exposure is considered a risk factor for sedentary behavior, for cardiovascular and metabolic diseases.^4^

Another aspect observed was that the pandemic may have contributed to an inadequate diet. During the COVID-19 pandemic, there was a 13.26% increase in the consumption of ultra-processed foods^11^ and a 48.58% increase in the consumption of frozen food, chocolates, sweets and packaged snacks.^4^ This may suggest that during the pandemic, adolescents, when staying at home, prefer to consume more frozen, fast, instant and industrialized foods, which may be related to their practicality and ease of preparation.^4,15^

*Concern with the healthy development of children is constant, but this moment of greater seclusion, socializing and management of time and household tasks by adults, who started to work at home, often result in a certain “relaxation” of the family diet, with quicker and easier solutions, such as ordering delivery food or replacing one of the meals with a snack, pizza or something similar*. (O Estado de São Paulo, São Paulo, Brazil, 07/23/2021 — Children’s food worries families on vacation and during the pandemic).

On the other hand, about 8% of adolescents^16^ stopped eating due to lack of money at home to buy food. The *Programa Nacional de Alimentação Escolar* (PNAE)^2^ is considered the most comprehensive and successful public policy in force. Created between the 1940s and 50s, it offers meals to more than 40 million Brazilian students from the public school system.^17^ The food safety of these adolescents depends on school meals, as explained by the newspaper *Zero Hora* (2020): “We know that there are still children who go to school mainly to have access to food”.

Brazilian school meals play a role of social protection, not only helping to eliminate hunger, but contributing to biopsychosocial growth and development, learning, school performance, as well as the formation of healthy eating practices, through food and nutrition education actions and the provision of meals that cover the nutritional needs of students during the period they remain in the school environment.^6^ All these practices were compromised during the pandemic and no policy to rescue these losses was directed to this population.

### Physical activity during the pandemic

Since before the pandemic, diseases linked to obesity, physical inactivity and lack of PA were already on an upward curve in society.^11^ It is noteworthy that despite all the knowledge acquired about the benefits that the practice of PA provides and its relationship with the improvement of QoL and MH,^4^ this subject was the least found in the initial search (n=09) (Table 3). Yet the need for confinement and social isolation, the limitations on circulation and PA suddenly imposed themselves on the lives of these adolescents.^17^

*The situation became more critical during the COVID-19 pandemic. Social isolation reduced physical activities, especially those done in groups. Time dedicated to sedentary activities, such as video games, cell phones and other screens, have become the only option for fun for most*. (O Liberal, Pará, Brazil, 08/01/2021 — 5 to 8% of children and adolescents in Pará are obese).

The reduction in PA practice and the increase in sedentary behavior among adolescents are also worrisome, given the association of these intervening factors with the risk of chronic diseases.^4,11^ In addition, most adolescents remained confined at home during the time of social distancing^4^ without the presence of outdoor activities and lack of interaction with friends, thus resulting in a decrease in sports practices, in PA time and in a worsening of sedentary habits.

Obesity is a multifactorial disease that can cause multisystem complications and affect several organs.^17^ There is an excerpt where the journalist analyzes a published article exposing the danger of a sedentary child or adolescent being contaminated by the COVID-19 virus.

*When obese children or adolescents are contaminated by SARS-CoV-2, these changes can complicate the situation, increasing the need for ventilatory support (when there is respiratory failure) and disrupting the immune response (defense of the body), among other possible events*. (Diário Gaúcho, Rio Grande do Sul, Brazil, 08/13/2020 — Study on obesity warns about children and adolescents in social distancing).

The regular practice of PA combined with changes in eating habits and the adoption of a healthier lifestyle can help in the prevention and treatment of obesity.^17^ In adolescents, this practice has a direct influence on physical and bone development.^18,19^ On the other hand, sedentary behavior increases the chance of cardiovascular risk, decreases energy expenditure and is generally associated with the consumption of high-calorie foods and soft drinks,^20^ in addition to having been shown to be a risk factor for adolescents who contracted COVID-19.^21^

### Mental health during the pandemic

Although MH was the category identified with the highest number of publications (n=42) (Table 4), it was observed over almost a year and a half of document analysis that much more could have been done for the health of adolescents in terms of journalistic coverage. There were reports of psychological suffering resulting from mental and emotional disorganization caused by the abrupt change in routine.

The COVID-19 pandemic, in addition to causing serious threats to the physical health of adolescents,^13,22^ has also triggered a wide variety of psychological problems such as anxiety and depression. The reports reflect the effects of social isolation and the experience of the pandemic on the MH of adolescents:

*Social support is one of the factors that most protect against depression in adolescents. According to experts, teenagers that already have a mental disorder were even more affected by the lack of school attendance during the pandemic*. (Zero Hora, Rio Grande do Sul, Brazil, 04/25/2021 — Young people face ‘cage syndrome’).

*The more anxious our society is, the more anxious the future ones will be. What will the development and future of a child or adolescent for whom we do not preventively address mental health look like? How will we deal with the impacts of the “cage syndrome” (fear of going to school and leaving home) aggravated among teenagers due to the pandemic?* (Folha de São Paulo, Brazil, 07/14/2021 — It is not a pandemic effect: mental health was already a public health problem and it is all of us).

The interruption of face-to-face school activities may be related to the anxiety rates found in adolescents.^15^ The school can act as a referral space for MH care services and also as a way to maintain routine habits, such as nutrition, for example, also functioning as an external support for adolescents in vulnerable situations.^23^

*It is necessary to clarify that life will be resumed with changes. This return will also require an ability to adapt to a new reality, not least because we are no longer used to life before the pandemic*. (O Globo, Rio de Janeiro, Brazil, 06/25/2020 — Depression increases among children and adolescents during the pandemic).

With the decrease in contact with the peer group, teenagers end up spending more time at home with feelings of loneliness and fear of the uncertainties of the pandemic. Associated with the worsening of psychological distress and social isolation, this may be associated with an increased prevalence of symptoms of post-traumatic stress disorder,^12^ correlated with other mental symptoms being directly proportional to the amount of time in isolation: the longer the time, the greater the prevalence of symptoms consistent with psychological distress.

*Extended isolation caused young people to withdraw for a long period from experiences that were intrinsic to their daily lives and to which they were already familiar. As a result, there was an increase in reports associated with heightened alert states, accompanied by some level of suffering. Anguish and avoidance in the face of new stimuli such as turning on the camera in online activities, complaints about fears that have already been overcome, infantilized behavior, excessive concerns regarding contamination by COVID-19 and resistance to returning to school can be reflections of a more intense state of anxiety* (Folha, São Paulo, Brazil, 07/22/2021 — Mental health when returning to face-to-face classes).

Adolescents and young people will be able to feel the impact of the COVID-19 pandemic on their health for many years to come and, according to the results of an international survey of adolescents and young people,^20^ one in five adolescents on average feels depressed or has little interest in doing things. This issue is directly related to the feeling of loneliness and distance from friends, the absence of professional counseling at student facilities, loss of freedom to come and go, concern for the future of the job market in which they will be inserted.^4,12^

Adolescents with significant levels of anxiety and depression felt helpless with regard to care for their MH. This feeling of helplessness may be related to specific MH practices that may have been paralyzed or difficult to access because of the situation or external supports such as friends, school and family members who do not live in the same house.^24–27^

### Pandemic and social vulnerability

The closing of several services, including schools, has had a negative impact not only on the right to education but also on other human rights, such as the right to quality food promoted by school feeding programs.^16,28^ With the pandemic, it is known that this cruel reality was considerably accentuated, expanding the social asymmetries that had already plagued the Brazilian population since its formation. Meanwhile, in public schools, where there is a shortage of material and human resources, the number of students who gave up studying multiplied.^29^

*The new coronavirus pandemic forced the suspension of face-to-face classes in schools around the world, which exacerbated inequalities among students. 2018, according to UNESCO’s 2020 Global Monitoring Report on Education. With the pandemic, inequality among students has worsened* (Correio Braziliense, Distrito Federal, Brazil, 03/26/2020 — Unesco: pandemic exacerbates the exclusion of children and young people to education in the world).

Among the different reasons for a disconnection from studies is the fact that many adolescents need to contribute to the family income and others do not have access to technological resources, which makes it impossible to monitor remote classes.^14^ In addition to this, millions of students, despite being regularly enrolled, did not receive guidance or were not able to adapt to carrying out school activities on their own in the distance modality.^16^ This inequality that underpins Brazilian society became more evident during the pandemic and the inequalities and differences between economic classes became prominent in the country to the point where we have a school dropout rate of 12% in the adolescent population.^3^

*And it’s not just work that many have had to give up, education is another field of abandonment. Young girls and adolescents, mostly black and from poor suburbs, had to leave schools to help at home. Housework is still one of the main goals for women. According to data from the Continuous National Household Sample Survey (Continuous Pnad), there are 7 million women who have returned to domestic work — which does not exist. A brutal throwback to decades of advancement*. (Folha, São Paulo, Brazil, 05/03/2021, The pandemic impacts maternal mental health and signs of stress and depression).

These adolescents are social actors represented and capable of identifying how reality impacts their lives and those of other people around them, thus building their own representations and narratives.^30^ Social representations are sets of practical knowledge of everyday life, collectively constructed from day-to-day challenges, imposed by phenomena that cause strangeness to subjects, altering their ways of thinking, feeling and acting.^30^

Despite identifying a situation of alert given the low social interest by the adolescent public expressed by the journalistic media, this research did not evaluate the long-term effect that exposure to isolation and its consequences can cause; in addition, document analysis should be used with caution in possible generalizations. Another possible limitation was the small number of publications found, as communication media did not pay due attention to the negative impact the COVID-19 pandemic had on the health of adolescents. However, the richness and complexity of the topic opens up future possibilities for continuity or expansion of different points of view. The results described here can support policies and strategies that are able to deal with priorities, but without the detriment of others. Pandemics and catastrophes can occur, but assistance plans for all populations can and must be developed to try to minimize any health problems.

In an expanded view, studies on communication, the media and health are necessary in order to foster a greater discussion around how information circulates and is appropriated by different audiences. The fact that adolescents do not present themselves as a mortality risk group contributed to this audience being placed in the background of media attention. It is important to debate what kind of news the population had access to during the COVID-19 pandemic and how this reflects, or not, the reality of that moment. It is in this context that studies such as this one can contribute to the area of public health and, specifically, adolescent health.

The authors conclude that the communication media did not adequately address the negative consequences that the COVID-19 pandemic had on the QoL, PA and MH of adolescents during the pandemic period, more specifically between 2020 and 2021. Of eight thousand publications that were related to adolescents and COVID-19, only 3% were about QoL, PA and MH. The newspapers viewed teenagers as excluded in the pandemic, with regard to journalistic priorities. The analyzed reports showed that the pandemic intensified the adolescents’ weaknesses, causing a decrease in social interaction, feelings of uncertainty, fear and the appearance/exacerbation of symptoms of anxiety, stress and depression. Social vulnerability was presented as another obstacle to be faced in this problem. In this sense, with the advancement of studies on and the understanding of long-term symptoms related to the disease, it is essential to continue research on this subject not only in health studies, but also in studies focusing on how to communicate and inform such issues.
